# "The physician as poet" review of: Pereira, Peter *Saying the World*

**DOI:** 10.1186/1747-5341-1-8

**Published:** 2006-05-17

**Authors:** Joel Weishaus

**Affiliations:** 1 Visiting Faculty Department of English Portland State University Portland, OR, USA

## Abstract

Peter Pereira is a family physician and a poet. I weave excerpts from Dr. Pereira's poems into a brief history of medicine's mythological and historical roots, beginning with the Egyptian god Thoth, and the Greek physician Hippocrates. Along the way, I touch on the European Middle Ages and the Islamic World. Finally, I quote poet-critic T.S. Eliot, who was an early influence on Dr. Pereira's decision to become a poet, and contemporary physician-poets Rafael Campo and William Carlos Williams. I end by placing Dr. Pereira, whose practice is oriented toward immigrant families, in his indigenous Pacific Northwest, arguing that being both physician and poet helps Pereira to live in a world that is both intimately human and naturally impersonal.

## The physician as poet

1.

Peter Pereira is a physician who has lived in Seattle, WA. for most of his life. He did his undergraduate work in both Biology and English, before attending the University of Washington Medical School. One of a growing number of doctors who balance the craft of medicine with poetry's intuitive tropes, Pereira begins this first collection with a poem titled "Nosophilia," which means "love of affliction." Why begin here? "How else count, recount what woes?/Pimples that burn. Fevers that shiver./Shinsplint. Bowleg. Backache. Rupture./What pains us makes us *us*." As the brain processes everything we call *us*, whether consciously or unconsciously, physical and emotional pain cannot be tweezed apart. One aspect, very palpable to a doctor, is loss. Because this state of emptiness can begin even before birth, Pereira initiates the body of his book with a poem titled "Fetus Papyraceous."

Sometimes one of the twins dies

in utero, without his mother

ever knowing she'd been twice blessed.

Hungry for life, the living twin

Will absorb his double...

The title refers to papyrus, a plant of the sedge family that ancient Egyptians, Greeks, and Romans soaked, pressed, and dried into thin slices of the resilient material on which they preferred to write. In Egyptian hierology, language and medicine were unified in the figure of Ibis-headed Thoth, "the lord of writing," from whom we also received astronomy, mathematics, philosophy, music, and the healing arts. Pereira writes that the unborn child, whom he calls "our paper twin," "remains a faint imprint/barely visible in the translucent web/of amniotic membranes – a fetal hieroglyph." So it begins here, with loss and recovery, written on "the blank page onto which all/our imagined lives are written."

In the poem, after his internship in which he saw a patient's "eyes rolling back and the sickly ashen color/of a face before death," the fledgling doctor is called to perform his "First Crash Cesarean."

Hold it like a wand, you say

as I guide the blade across shaved skin,

into layers of yellow fat and fascia

stained crimson.

A school of hermetic learning gathered around Thoth, beginning in Phoenicia, then moving to several islands off Asia Minor, finally settling in Ionia, where the legend of Aesculapius holding the herald's wand already thrived. There are two staffs: Aesculapius' has a single serpent, "the dragon of Media" wound around it, while the staff of Hermes (Hermes-Thoth, who later became known as Hermes Trismegistus) is the caduceus, had two serpents. Both became a symbol of the medical profession, the caduceus by the more circuitous way of alchemy.[1]

Aesculapius means "unceasingly gentle." He is pictured as a wise old man. How wonderfully this echoes the general concept many of us have of our family doctor. Mine was named Solomon. Whether Dr. Solomon was wise, I don't know. But to my family he was somewhat like a god, possessing arcane knowledge that shepherded us safely through dark valleys of illness. In "Baby Made of Flowers," Dr. Pereira echoes the biblical Solomon, reminding us that "even a baby who is dead must still be born." Although the paternity of this child, whose "two blue feet/took their only steps across a sheet of paper," wasn't in question, the mother was "a bleeding woman with no baby/to feed, to comfort." There are many ways of being denied. All through this book, even when facing death, Pereira's world is sensual. In "Winterbloom," he writes:

The knot in your breast was already

the size of a garlic clove...

Then we descended a damp trail

to where a Chinese witch hazel

that day burst into bloom...

filling the air with a sweet

faintly medicinal fragrance.

After suffering through rounds of chemotherapy, she thought she was rid of the cancer. Then, like the returning of her amber-red hair, the tumor reappeared. Now the poet walks the winter garden alone, the witch hazel "in bloom again/it's red-orange tatter/a shimmering confetti, its citrus-musk/a bitter sweet.

Aesculapius had two daughters: Hygieia, known now as Preventive Medicine, and Panacea, the mythical cure-all in our age of vaccinations. Hygieia and Panacea are indeed sisters. But from Athena, the warrior-goddess who guided Odysseus' perilous journey home, Aesculapius had received the "Gorgon's blood," a magical homeopathic serum that could either kill or resurrect the dead, depending on the physician's will and skill. When Aesculapius used the substance to resurrect a patient, Hades, Lord of the Underworld, was angered by the doctor's arrogance. He complained to Zeus, who agreed. All physicians painfully must learn that there is a time to let the patient go. With one of his famous lightning bolts, Zeus sent the soul of the patient back to Hades, and Aesculapius into the sky as a constellation. "Take care, we say to one another, on parting," wrote Dr. Pereira, "as if the cargo/we carried were fragile/or dangerous---."

The historical Father of Medicine, Hippocrates, was born on the island of Cos. Little is known of his life or medical training, but to this day physicians observe signs of death's approach as Hippocrates described them: Dry skin, sunken eyes, "sickly ashen" color, sharpened features; especially the nose, which becomes "Sharp as a pen," Shakespeare observed. Breathing out its last words, Pereira ends a poem titled "What Matters" –

We kiss your face, squeeze your hand;

hang on before we let go.

Hermes continued Thoth's many endeavors, inventing the lyre and the flute, which he gave to Apollo in exchange for a golden caduceus. Though there had been some brilliant physician-writers, like Galen, born 130 A.D. in Asia Minor, who followed Hippocrates' lead, the medical profession "survived in western Europe between the seventh or eighth and the eleventh centuries mainly in a clerical or monastic environment," an environment in which it was sometimes "difficult to distinguish between physical healing and spiritual counsel and the encouragement in the help offered by the clergy." [2] Many of the medical treatises, especially philosophical ones stemming from the Greek, were taken up by the Islamic World. In part, it was Nestorian Christians, persecuted by Rome, who had delivered the precious knowledge to the Middle and Near East; in fact, "specialist medical research, free public health treatment and retirement homes for the aged were available in Baghdad and other parts of the Islamic world in AD 950, while Britons were still in the Dark Ages."[3] Strangely, we know of many of the great Arabian physicians because of the English poet Geoffrey Chaucer, who was the leading scholar on "Mohammedan" medicine of his day. His *Canterbury Tales *lists them, along with other notable physicians, like "old Esculapeus,/and Descorides and Rufus too;/add Hippocrates and Haly and Galien/Serapyon, Razis, and Auycen..."

2.

In an email to me, Dr. Pereira wrote: "When work or love or life are a little too wild and out of control, turning to write about it is a way to contain the intensity, to hold it at arms length, and rotate it in space and see it from all sides, and come to a deeper understanding, make it manageable, survive it. Conversely, if I am feeling disconnected, or numb, or distanced, writing poetry is a way to access the realm of feeling and emotion, and reconnect with the world." Pereira went on to say, "Some of the poets whom I admire, and who have influenced me: T. S. Eliot was an early favorite. I remember my high school English teacher reading 'The Love Song of J. Alfred Prufrock' to the class, and having an incredibly intense reaction to it. One where it feels like the top of your head is lifting off, and the poet is speaking directly to you. And I wanted to write a poems like that. I went out and read everything I could find by T. S. Eliot."

Let us go then you and I,

When the evening is spread out against the sky

Like a patient etherized upon a table...

To the would-be doctor, these lines would have been more like a door than a table. However, Eliot's theory of "depersonalization" would not appeal to a fully fledged doctor, especially one in clinical practice. In his essay, "Tradition and the Individual Talent," Eliot wrote that "art may be said to approach the condition of science." Then he uses an alchemic analogy in which "the action takes place when a bit of finely filiated platinum is introduced into a chamber containing oxygen and sulphur dioxide," which forms a sulfurous acid. "This combination takes place only if the platinum is present; nevertheless the newly formed acid contains no trace of platinum, and the platinum itself is apparently unaffected; has remained inert, neutral, and unchanged."[4] Eliot remarks that "the poet has, not a 'personality' to express, but a particular medium, which is only a medium and not a personality, in which impressions and experiences combine in peculiar and unexpected ways." Like science, Eliot's philosophy of poetry triumphs over psyche. Yet, by the mid-1950s, not long after Werner Heisenberg famously included the observer in his equations, a new school of confessional poets, the Beats, loudly announced that the psyche, like Pan, had returned.

The term "confessional," as it applies to Modern Poetry, was coined by M.L. Rosenthal in 1959, with Robert Lowell's book *Life Studies *in mind.[5] Though nothing like it in tone or technique, Lowell's prize-winning book was inspired by Allen Ginsberg's monumental rant at post-World War II middle-class America, "Howl." Ginsberg went on to the public stage literally and figuratively undressing himself, while confessing his homosexuality. In brilliantly-crafted scatological riffs, he pioneered the way for poets who, like Pereira, are gay, but have a less confrontational, quieter, nature. In, "The Boy Who Played With Dolls," Pereira remembers himself at around age five playing with two large naked dolls, their "hair shorn, arms and legs akimbo/as they dangle from his hands."

Years later, his mother will say:

You weren't a sissy, you were practicing

*to be a doctor*.

In "Kafka's Grave," Pereira mourns the death of his brother, "a military man/who disappeared without leaving anything/written." Noticing family resemblances in his orphaned nephew, he writes that "a stranger/would think he is mine, yet/I'll surely never be called father/or husband by anyone in this life...I will never author a living child." Instead, the poet "fill(s his) pen with ink," a Freudian flourish, "and wander(s) out to the wooden bench/under the flowering plum to write." In the garden, he takes comfort in the fact that "Kafka died childless,/a bachelor surrounded by books/he never saw published." Yet, because Kafka's stories live and propagate in minds through the ages, "his grave's/alive with loving flowers."

3.

During the Middle Ages and into the Renaissance Western Medicine included in its pharmacopoeia charms, amulets, and prayers. Rafael Campo, a Harvard Medical School physician-poet, writes of this time: "the body itself was 'read' or interpreted as a text, and manuscripts meditatively produced by monks contained such 'prescriptions' as exhortations against the sinfulness that was thought to cause disease, and specifically linked to prayers for healing."[6] While in clandestine laboratories alchemists boiled and toiled various substances in flasks, retorts, alembics, thinning out the irony of a materialistically-based science whose quest was covertly spiritual. Besides their experiments, which led to modern chemistry, "alchemy gives us a language of substance which cannot be taken substantively, concrete expressions which are not literal. This is alchemy's therapeutic effect: it forces metaphor upon us."[7]

In his email to me, Pereira went on to say that, "Because of its densely compact language, metaphorical content, and synthesis of disparate pieces of information – images, mood, tone, sensory details – the reading and writing of poetry keeps me in touch with my more intuitive and irrational side, and I hope makes me a better listener, to get a closer, more accurate read on patients and their stories." He comes up on the other side of Susan Sontag's Platonic argument that, "the most truthful way of regarding illness – the healthiest way of being ill – is one most purified of, most resistant to, metaphoric thinking."[8] But any good doctor can tell us that a case history not only consists of scans, blood tests, and listening to the pulses of the body. It is also about listening closely to the patient's personal account, listening to the tone of the voice, to the metaphors behind the complaint.

Pereira, whose practice serves a poor community that consists of many immigrants, sometimes must collect the case history of a patient through an interpreter. In poem, "What is Lost," relates the story of a Cambodian woman who "came across the border" with "no shoes---only one black Cambodian skirt,

a thin blouse, the long

scarf they use for everything: sleeping,

bathing, carrying food, wrapping

the bodies of the dead.

She asks the doctor if he has anything "to keep the nightmares away," while refusing to speak of the past that returns in her dreams, "afraid if words bring (her husband and brothers) back, along will come the soldiers." As the translator works his English into the foreign tongue, Pereira wonders "which sound was nerve, which/was heart, which grief."

Of physician-poets there were a few who chose to leave the medical profession in order to write full-time. Perhaps the best known of these is John Keats, who, until recently, wasn't given much credit for his early training as an apothecary, the 19th Century's equivalent of a present-day family practitioner. His "Ode on Melancholy" begins as if he were instructing someone in the preparation of a medicinal substance –

No, no, go not to Lethe, neither twist

Wolf's-bane, tight-rooted, for its poisonous wine;

Nor suffer thy pale forehead to be kiss'd

By nightshade, ruby grape of Proserpine;

Make not your rosary of yew-berries...

Pereira's list of poets who have influenced him begins with T.S. Eliot, but does not include Eliot's contemporary, family physician William Carlos Williams. However, I think it is safe to assume that he has read Dr. Williams, as Williams is the most often cited example of the physician-poet of the 20th Century. Unlike his prose, Williams' poems don't give many clues to the fact that he was a practicing physician. In his book, *Autobiography*, he wrote of "the thing" – which he doesn't name, but I take for the human spirit – and how he was permitted by my medical badge to follow the poor, defeated body into those gulfs and grottoes. And the astonishing thing is that at such times and in such places---foul as they may be with the stinking ischiorectal abscesses of our comings and goings – just there, in all its greatest beauty, may for a moment may be freed to fly for a moment guiltily about the room.[9]

On the other hand, T. Hugh Crawford, in *Modernism, Medicine, & William Carlos Williams*, concludes that "what has emerged in this discussion of Williams and modern science has been a concern for objectivity (and objectification), clarity, efficiency, and cleanliness.'[10] While Williams' poems clutch the Modernist/Cartesian model, many of his successors not only draw their poems from their examining rooms and surgeries, but go into the mundane world with their black doctor bags worn like backpacks. (I think here of the distinguished poet Robert Creeley, who said that he became a writer in part because, like his physician father, who made house calls, he wanted to be able to carry all the instruments he needed for his profession a single bag.)

A denizen of the Pacific Northwest, Pereira is not only comfortable with the latest medical technologies, but also with taking with the pulse of a mountain. He begins "Hiking to Tsagaglalal Petroglyph, Thinking of Guy Anderson at 90" –

High above the valley, where midday

sun makes a kiln of treeless rimrock, I stand in supplication before her. Mother of everything, mouth drawn by the centuries her long-ago composed eyes keep watch as the Columbia gorges its path to the Pacific.

Like Williams, Pereira feels an affinity with painters, remarking here on how the regional artist Guy Anderson's work evolved from "angular clutter and affliction" to "the soft/rounded sumptuousness of male and female nudes/drifting above egg-like spheres, each one tethered/to a wide oceanic void by an umbilical streak of red," while scaling the basalt cliffs that give way "to bare rolling steppes, the clefted sinews/of the basin's great unfinished canvas."

Pereira approaches nature not as a healer, but, as he says in the title poem, which is also the last poem in this book, "Intent/only upon the corn's silk/streaming in the breeze, a honeybee/darting about the pink geranium. The depth of his poetry lies in his acceptance of the natural world as sensually impersonal milieu; while his practice as a physician requires intercessions on an intimately engaging level. This dichotomy creates a fulcrum on which he's able to balance these two demanding endeavors. "Stand with me for a while," he concludes,

Imagine the silver lily returning

before we say goodnight.
